# Nile Crocodiles in Lake Nasser, Egypt, Are Found Close to Fishermen's Camps, Indicating Potential Conflicts

**DOI:** 10.1002/ece3.71970

**Published:** 2025-09-02

**Authors:** Mohamed A. Ezat, Elke Molenaar, Marc Naguib, Frank van Langevelde

**Affiliations:** ^1^ Behavioural Ecology Group Wageningen University & Research Wageningen the Netherlands; ^2^ Wildlife Ecology and Conservation Group Wageningen University & Research Wageningen the Netherlands; ^3^ Aswan Protectorates Office, Nature Conservation Sector, Egyptian Environment Affairs Agency Aswan Egypt

## Abstract

Conflict between wildlife and humans is one of the main causes of wildlife decline. Numerous studies have investigated environmental and anthropogenic variables determining the distribution of large carnivores to predict and mitigate the risks of such conflicts. However, for aquatic carnivores, such as crocodiles, little is known about which variables explain their distribution. Yet, human–crocodile conflicts are on the rise. A better understanding of such variables will potentially prevent conflicts or even promote coexistence between crocodiles and humans. Here, we analyze which environmental and anthropogenic variables determine the distribution of Nile crocodiles ( *Crocodylus niloticus* ) in Lake Nasser, Egypt. As apex predators, Nile crocodiles are often perceived to be responsible for the declining fish populations, and proximity to fishermen could lead to conflicts and killing of crocodiles. Since both crocodiles and fishermen hunt fish, we expected to find Nile crocodiles close to fishermen's camps. To analyze the crocodile distribution, we surveyed 1880 km of Lake Nasser's shoreline and collected 192 sightings of Nile crocodiles. We used readily available spatial data for 23 environmental and anthropogenic variables, including ambient temperature, the slope of the shoreline, and the distance to fishermen's camps. We used MaxEnt species distribution modeling to quantify which variables were correlated with Nile crocodile locations. Our analyses revealed a higher probability to find Nile crocodiles closer to fishermen's camps. Additionally, crocodile presence was positively associated with flat shorelines. The findings that crocodiles are not driven away by fishing activities reflect a conflict between fishermen and crocodiles. This study contributes to an understanding of which environmental and anthropogenic variables determine the distribution of Nile crocodiles, a key conservation point to promote human–crocodile coexistence.

## Graphical abstract

We investigated the relative importance of environmental and anthropogenic variables determining the distribution of Nile crocodiles to predict and mitigate the risks of human–crocodile conflicts. A better understanding of such variables will potentially prevent conflicts or even promote coexistence between crocodiles and humans. We found a higher probability to find Nile crocodiles closer to fishermen's camps. Additionally, we showed that crocodile presence was positively associated with flat shorelines. The findings that crocodiles are not driven away by fishing activities reflect a looming conflict between fishermen and crocodiles.

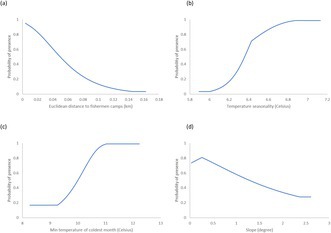

## 1 Introduction

Apex predators play a vital ecological role in their ecosystem as they shape lower trophic levels, and are often implicated in human–wildlife conflicts (Estes et al. 2011; Ripple et al. 2014; Everatt et al. 2019; Somaweera et al. 2020). Indeed, the majority of apex predators compete with humans for the same resources and habitat. In the Anthropocene, human–wildlife conflicts have pushed most species of apex predators into a conservation crisis (Treves and Karanth 2003; Estes et al. 2011; Ripple et al. 2014). For example, African lion ( *Panthera leo* ) populations have declined as a result of conflicts with humans (Everatt et al. 2019). Furthermore, the conservation of apex predators faces challenges, in many cases due to a lack of understanding of their distribution and density. This is especially the case as anthropogenic pressures pose negative effects on the available suitable habitats of apex predators, thus decreasing their abundance (Woodroffe 2000; Balaguera‐Reina et al. 2016; Everatt et al. 2019; Isberg et al. 2019).

Whereas most of the apex predators are often charismatic species for conservation and therefore well‐studied, other apex predator species, such as crocodilian species, are cryptic, and little is known about the effects of human activities on their distribution and density. Studies have shown that the correlation between crocodile density and human activity is a function of human–crocodile conflict, which varies according to crocodile species, habitat suitability, and level of human activities (Shaney et al. 2019; Ouedraogo et al. 2022). However, the association between crocodile presence and anthropogenic activity is complex as is illustrated by a study of the Nazinga Game Ranch in Burkina Faso, where West African crocodiles, *Crocodylus suchus*, were present at high densities in water bodies with and without fishing activity (Ouedraogo et al. 2022). Crocodiles may indeed be found in both undisturbed areas and those with high fishing activity, provided that the habitat is suitable with sufficient fish. Yet, this study of the Nazinga Game Ranch also showed that fishing activities had negative effects on crocodiles, such as injuries and the capture of small crocodiles by fishing nets (Ouedraogo et al. 2022). Moreover, the abundance of the saltwater crocodiles, *Crocodylus porosus* , and false gharials, *Tomistoma schlegelii* , in a river system in Sumatra in Indonesia negatively correlated with the proximity to humans and the presence of fishing traps (Shaney et al. 2019). The correlation between crocodiles and fishermen may depend on the available habitat and fish density (Shaney et al. 2019). At high fish densities, crocodiles may be more tolerant to fishermen's activities, while we expect that high levels of conflict may occur when fish densities are low and crocodiles leave highly disturbed areas. The latter leads to a negative correlation between crocodiles and fishermen. In areas where there is a positive relationship between fishing activity and crocodile presence, humans may perceive the crocodiles as a threat or strong fishing competitor, leading to an increase in human–crocodile conflicts (Shaney et al. 2019; Ouedraogo et al. 2022). Moreover, if the available habitat, especially along shorelines, is limited, we expect that crocodiles may have fewer opportunities to avoid areas with fishermen. Thus, identifying the nature of the human–crocodile conflicts lies in first identifying the variables affecting crocodile distribution (Parker 1970; Focardi and Cagnacci 2012; Calverley and Downs 2014; Pooley 2015; Sai et al. 2016; Shaney et al. 2019; Ouedraogo et al. 2022).

In this study, we determined the relative importance of anthropogenic and environmental variables that can explain the distribution of the Nile crocodile, *Crocodylus niloticus* , in Lake Nasser in Egypt. In this area, a growing human population is combined with high fishing activity (Bishai et al. 2000; Shirley et al. 2012). The Nile crocodile population in Egypt is classified as “Least Concern” according to the International Union for the Conservation of Nature (IUCN) Red List (IUCN 2007). However, since the early part of the 21st century, the growing illegal trade in crocodiles from Lake Nasser has underscored the urgent need to institute sustainable management policies for this species (Isberg et al. 2019). The Nile crocodile often inhabits wetlands that are under local threat, and this species is considered an important indicator of the environmental conditions (Mazzotti et al. 2009). Thus, a better understanding of the Nile crocodile distribution based on environmental and anthropogenic variables in Lake Nasser is a key conservation point to better understand human–crocodile conflict and to potentially promote human–crocodile coexistence, in which both are coadapted to share the same space (Carter and Linnell 2016; König et al. 2020). Crocodiles are thought to cause extensive damage to fishing nets and to deplete the fish stocks, although this has been subject to debate (Somaweera et al. 2020). Moreover, the Nile crocodiles of Lake Nasser are thought to compete with local fishermen as they both harvest fish. The local crocodile population, therefore, has become increasingly threatened as many individuals are killed every year, often by local fishermen. Nonetheless, Lake Nasser still has a large, yet decreasing, Nile crocodile population, giving unique opportunities to study their ecology and behavior and the way they respond to human disturbance.

Here we mapped the locations of all crocodiles sighted and fishermen camps along 1880 km of Lake Nasser's shoreline. This allowed us to relate crocodile presence to the distance of fishermen’s camps. Although we anticipated the co‐occurrence of fishermen camps and crocodiles in areas with high fish densities, we predicted that the presence of fishermen camps would be negatively related to the presence of crocodiles. This negative relationship is attributed to the crocodiles' avoidance response, leading them to stay away from prime feeding grounds near fishermen's camps. We also expected Nile crocodile presence to be positively associated with flat shorelines.

## 2 Material and Methods

### 2.1 Study Area

**FIGURE 1:**
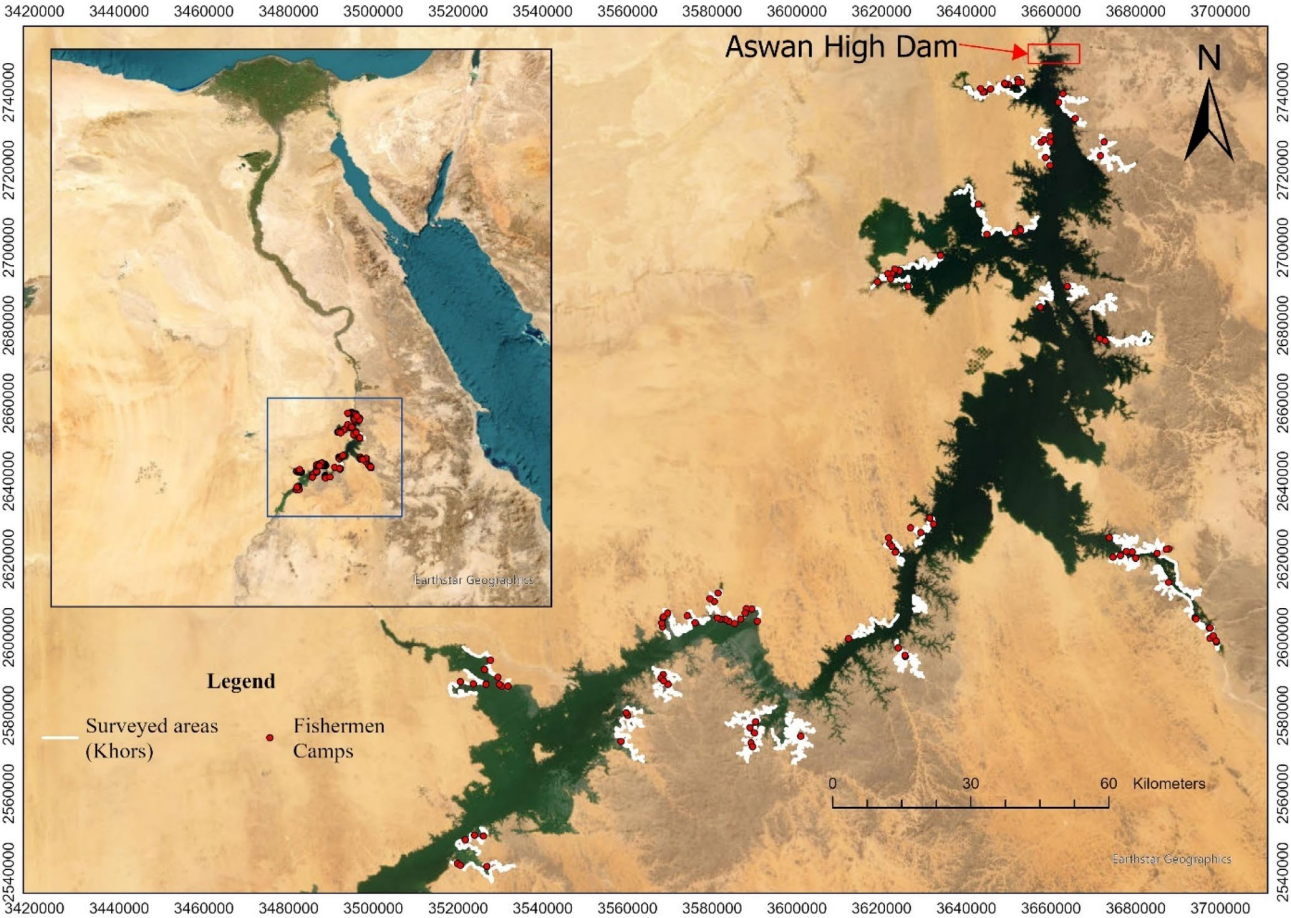
Surveyed areas (Khors) and the location of the fishermen camps in Lake Nasser, Egypt (background is the basemap that can be accessed from ArcGIS Online).

We investigated the distribution of the Nile crocodile in Lake Nasser, which was created with the construction of the Aswan High Dam across the waters of the River Nile between 1958 and 1970 (Figure 1). Lake Nasser is one of the world's largest man‐made lakes, with a seasonally and annually varying shoreline of up to 7844 km, branching into a network of many small side valleys (also referred to as khors). Approximately 6000 fishermen live in 750 camps spread around the lake, with each accommodating between 2 and 16 people (Bishai et al. 2000; Halls et al. 2015). Lake Nasser's shoreline can be divided into two main habitat types: sandy beaches and rocky cliffs. Sandy beaches are characterized by gently sloping shores composed predominantly of sand and loose rocky slate or gravel. Rocky cliffs are areas with sheer rock faces descending into the water with few or no haul‐out sites (Shirley et al. 2012).

### 2.2 Data Collection

#### 2.2.1 Nocturnal Spotlight Survey

To test the relative importance of anthropogenic and environmental variables determining the Nile crocodile distribution, we used nocturnal spotlight surveys (Parker 1970; Hill et al. 1987; Hutton and Woolhouse 1989). In 2018, we conducted systematic overnight crocodile counts using small boats and hand‐held spotlights (3 million candle high‐power lamp; Brinkmann Big, 800–2660‐1), along 1880 km which represented around 30% of the lake's shoreline (Bishai et al. 2000). The lake has five fishermen associations, each occurring in a different part of the lake with different numbers of boats and fishing licenses. We surveyed about 30% of the shorelines of each association area, which included 19 khors. We randomly selected a point on the shore of one of the 19 khors that we surveyed, and then split into two teams of researchers that moved in opposite directions along the shore. The survey lasted for 20 nights from August to October 2018, and each team covered approximately 45 km of shoreline per night. The survey started 1 h after sunset and continued for about 5.5 h each night (Figure 1). Crocodiles were detected through their eye reflections during the spotlight survey (Levy 1991; Montero et al. 2019). The spotlight survey was conducted by boats with an average speed of eight km per hour, along the shoreline and about 50 m from the shoreline.

#### 2.2.2 Environmental Variables

In total, we used 23 environmental and anthropogenic variables (Appendix 1), using a digital elevation model (DEM) and data on 19 bioclimatic variables from the World Climate Database (https://www.worldclim.org). The bioclimatic predictors contain current information on ambient temperature and precipitation interpolated from climate data for the years1970 to 2000 (Fick and Hijmans 2017). These predictors are broadly viewed as biologically meaningful to define the eco‐physiological tolerances of species (Graham and Hijmans 2006; Hu et al. 2020).

**FIGURE 2:**
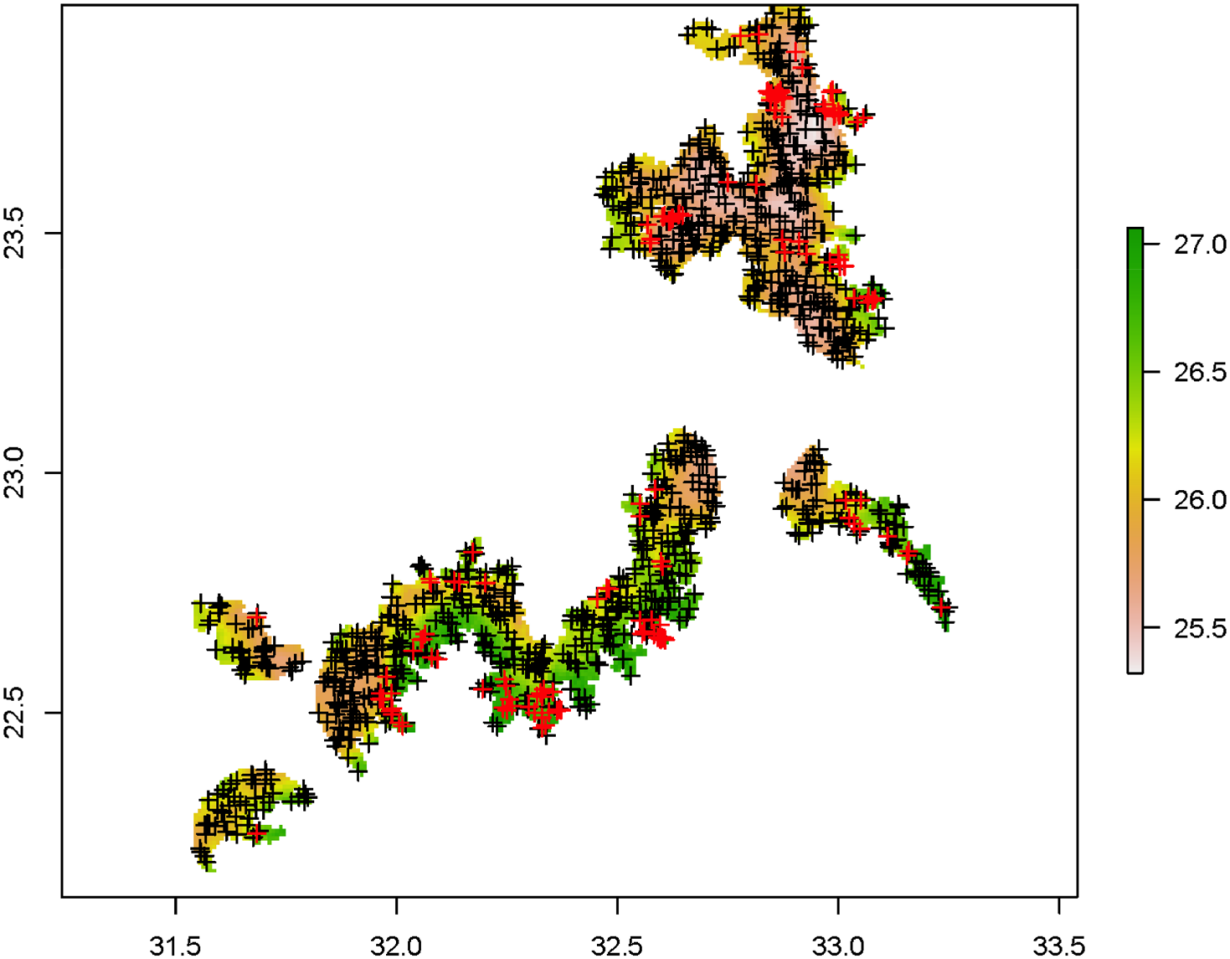
Example of the pseudo‐absence data (black plus) and presence data (i.e., crocodile locations found through the survey; red plus) plotted in the study area Lake Nasser with a buffer of 1 km inland are shown in red. The background layer represents the annual mean temperatures (bio1 from Worldclim at a resolution of 30 arc‐seconds).

All predictor variables were collected at a resolution of 30‐arc sec (or 1 km^2^) and clipped to the extent of Lake Nasser plus 1 km inland (Figure 2). We included the land within a buffer of 1 km (i.e., 1 grid cell) away from the shore because of the lake's water level fluctuations, and crocodiles can move inland. From the DEM, we calculated the slope for each grid. Additionally, to assess the relationship between environmental and anthropogenic variables and the Nile crocodile distribution, we determined the Euclidean distance to fishermen's camps to the center of each grid cell. We also derived the path distance that crocodiles would swim from the center of each grid cell to each fisherman's camps. The environmental variables, among others (Appendix 1), included different measures of ambient temperature (seasonality temperature [coefficient of variation of monthly temperature], minimum temperature of warmest month, minimum temperature of coldest month, maximum temperature of warmest month, and annual mean temperature). These measures for ambient temperature were used to control for possible effects, but they were not the focus of our analysis.

### 2.3 Data Analysis

We applied a presence‐only analysis (Phillips et al. 2006) due to the cryptic nature of crocodilian behavior. A frequently adopted method to determine a species' presence is MaxEnt based on maximum entropy. MaxEnt is a machine learning algorithm that models the probability of species occurrence as a function of independent variables, using presence‐only and pseudo‐absence data. Pseudo‐absence data are randomly selected points from the study area that represent the background conditions. The MaxEnt approach relies on available information on local conditions in which a species is present and establishes a model with maximum entropy based on that knowledge (Phillips et al. 2006). We used the MaxEnt species distribution model in R version 4.2.1 (https://r‐proj.org) using the package *dismo* version 1.3–9 (Hijmans et al. 2017). Both the species occurrence data and the 23 environmental and anthropogenic variables were used to build the model.

In MaxEnt, 10 replicates were calculated, using 50% of our occurrence data to train the model and the other 50% for testing the model's performance. 1000 random pseudo‐absence points were taken for the model runs but made reproducible using the static seed via the *set.seed* function. The results of the model were reported according to the default format, in which the predictive accuracy of the model was assessed by taking the Area Under the Curve (AUC). This value ranks between 0 and 1, in which any outcome higher than 0.5 is indicative of an ability to distinguish between training and testing data (Araujo et al. 2005). Before running the final model, we first tested the correlations between the independent variables as their effect in the presence‐only analysis might be less visible when they are correlated. Some of the independent variables were highly correlated. Hence, we used the permutation importance values from the MaxEnt model to indicate the relative importance of each independent variable in the model as the permutation importance value is less sensitive to collinearity.

## 3 Results

**FIGURE 3:**
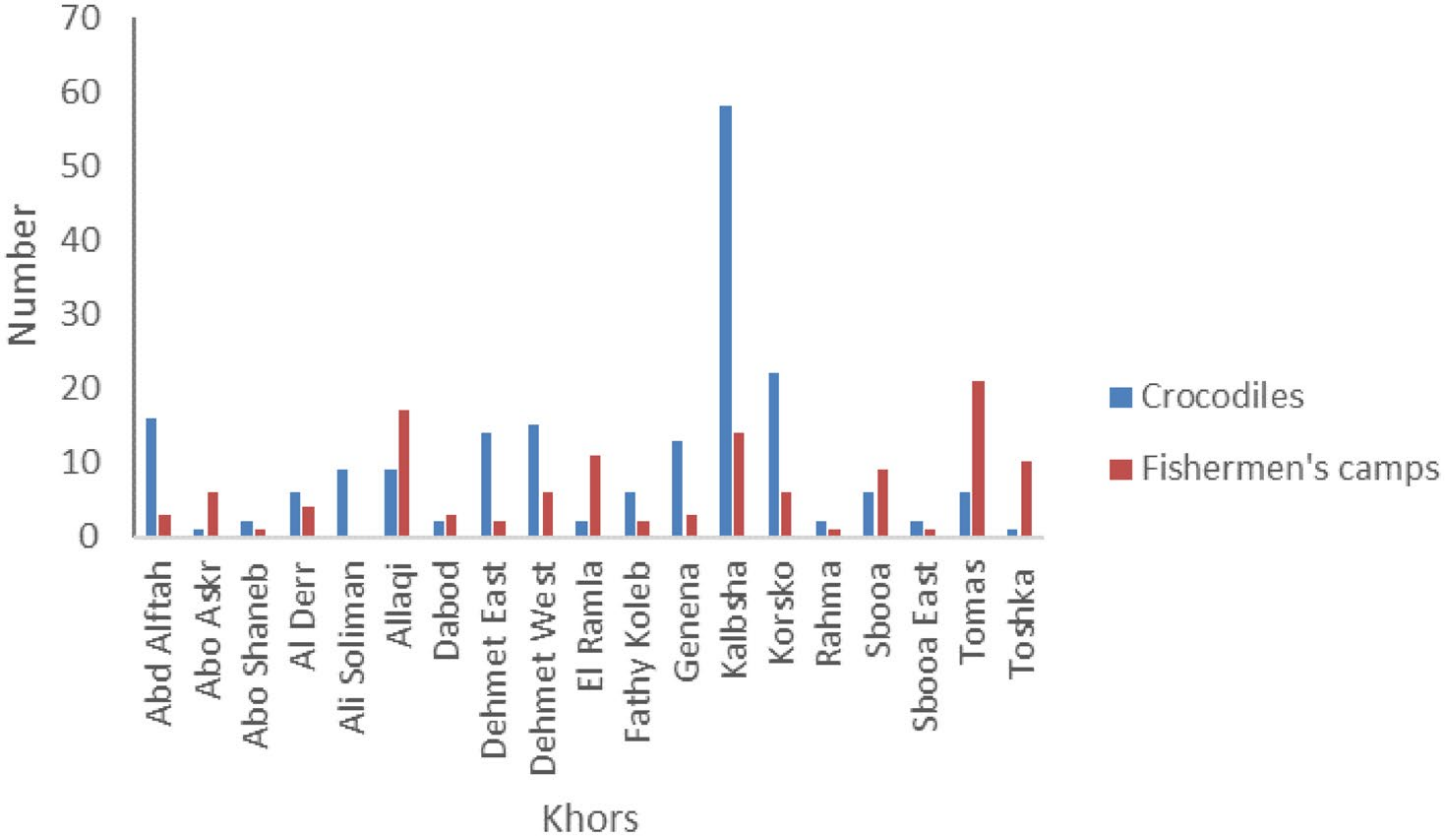
Number of Nile crocodiles and fishermen's camps in the different khors of Lake Nasser that we surveyed.

**FIGURE 4:**
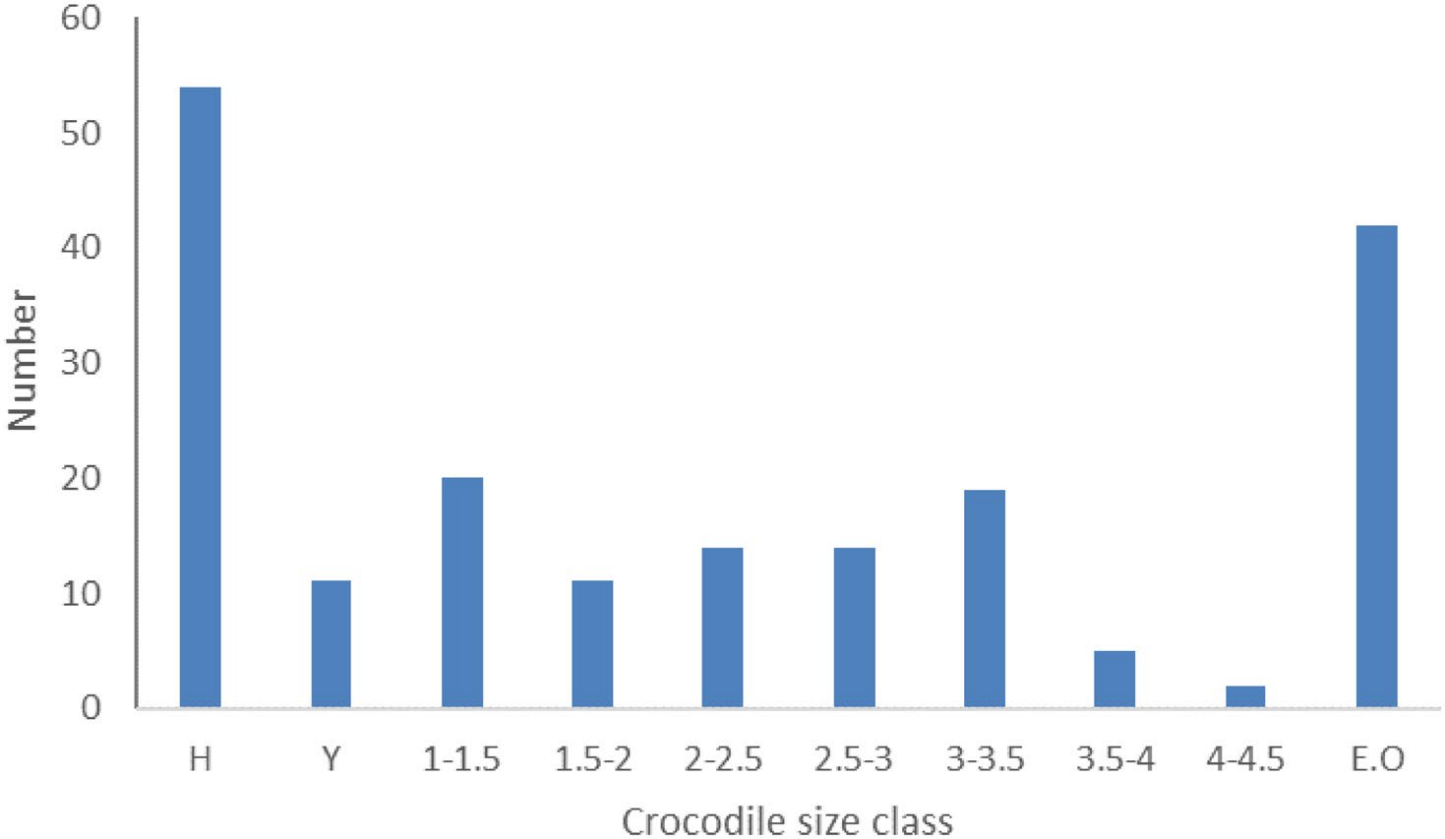
Size classes (in m) of the crocodiles that we observed: H = hatchlings, Y = Yearlings (< 1 m), and EO = “Eyes Only” for crocodiles for which a size could not be determined.

A total of 192 Nile crocodiles were observed during the spot‐night survey. We found crocodiles and fishermen's camps in each of the 19 khors that we surveyed (Figure 3). Among the crocodiles, 54 individuals were hatchlings (Figure 4). As we did the survey during the season when the young crocodiles hatch and adults do the parental care (Combrink et al. 2016), we assumed that the presence of several hatchlings in one observation indicated the presence of at least one adult crocodile. Hence, we included 146 unique locations for crocodile presence in our analyses out of 192 observations, reducing observations with multiple crocodiles to presence locations.

The results of MaxEnt showed the most important variables explaining the distribution of the Nile crocodile by using the permutation importance (Appendix 2): the Euclidean and path distances to the fishermen's camps, the seasonality temperature, the minimum temperature of the coldest month, and the slope of the shoreline. The permutation importance measures the decrease in model performance (e.g., AUC) when the values of a variable are randomly permuted. This measure is less biased by correlations between independent variables than the percent contribution. The average test AUC for the replicate runs of the model was 0.854, and the standard deviation was 0.070.

**FIGURE 5:**
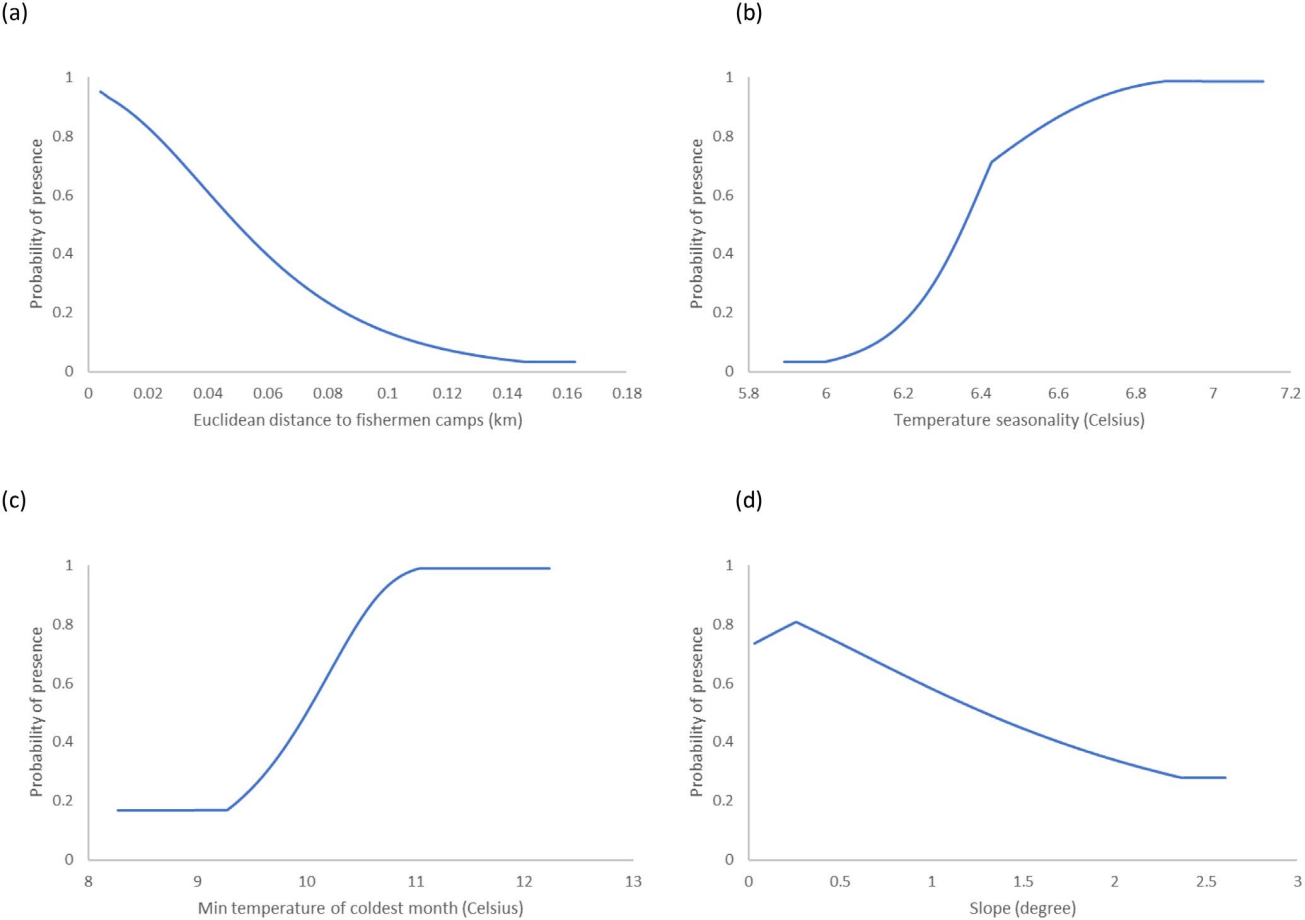
Different response curves of the Nile crocodile distribution from the MaxEnt model: (a) Euclidean distance to the fishermen's camps, (b) temperature seasonality, (c) minimum temperature of the coldest month, (d) slope of the shoreline.

The results show that the distance to the fishermen's camps, seasonality, temperature, and minimum temperature of the coldest month had a positive association with the crocodile presence in the lake (Figure 5a–d), while the slope of the shorelines had a negative association with crocodile presence (Figure 5e,f). In other words, crocodiles were more common near fishermen's camps than farther away, and in locations with flat slopes. All the estimates of the relative contribution of all the variables of the MaxEnt model are listed in Appendix 2.

## 4 Discussion

Here, we show that Nile crocodile presence was positively associated with the distance to fishermen's camps, i.e., the probability of detecting Nile crocodiles was highest near fishermen's camps. Moreover, Nile crocodiles were more likely to be seen at flat slopes compared to steep slopes, where the flat slopes are generally associated with sandy beaches and the steep slopes with rocks. The distance to the fishermen's camp was the main anthropogenic variable determining the Nile crocodile distribution in Lake Nasser.

The positive association between the crocodile presence and fishermen's camps contrasts with our prediction that crocodiles would avoid disturbed areas with human activities like fishing. These findings reflect a conflict between fishermen and crocodiles, most likely resulting from crocodiles foraging in the same areas as fishermen do, despite the risk of doing so. This supports the hypothesis that crocodiles and fishermen indeed compete for the same area in search of fish (Chakanyuka and Utete 2022). Moreover, the findings using the actual distance to the fishermen's camps as a variable expand on previous studies on different crocodile species in Burkina Faso. While earlier research had categorically related crocodile density to the presence or absence of fishing activity in different water bodies (Ouedraogo et al. 2022), this study unveils that a relationship within the same water body exists on a continuous scale. The findings, however, contrast a study on the occurrence of saltwater crocodiles and false gharials related to human settlements and fishing traps in a river system in Indonesia, where crocodiles were more likely to be seen in undisturbed areas (Shaney et al. 2019). Such negative correlations may either reflect high human pressure causing crocodiles to retreat from suitable habitats or a large area of suitable habitat such that crocodiles can avoid disturbance without incurring high foraging costs. The situation in Lake Nasser is different as there are no human settlements beyond the multiple small fishermen camps in almost the whole of the lake's khors (Bishai et al. 2000; Shirley et al. 2012). These findings suggest that there is almost no way for crocodiles to avoid these fishermen's camps, despite the large size of Lake Nasser. One reason may be that fishermen's camps are located on the flat shorelines in the khors, presumably allowing better access to more effective fishing grounds than the open waters in the central parts of the lake. Likewise, the shorelines of the khors have many sandy and flat slopes and a complex meandering shoreline, so it may be overall more suitable as crocodiles can find hidden places to rest and bask during the day. Hence, the suitable locations for fishermen's camps overlap with the suitable habitat that the crocodiles use for basking. Our results thus overall support the hypothesis that the correlation between Nile crocodiles and fishermen's camps depends on the available resting habitat.

Indeed, our findings that crocodiles were mostly seen at flat slopes of the shorelines are not surprising because these are the most easily accessible sections and sandy beaches are generally found in these locations. For conservation purposes, these results are important, as the presence of sandy flat shorelines varies with the water level (El‐Shirbeny and Abutaleb 2018). At lower water levels, there are usually more sandy areas available for nesting, basking, and resting. Thus, crocodiles might be more restricted in finding locations on land at high water levels. To fully unravel the relationship between suitable locations and the highly seasonally and annually fluctuating water level of Lake Nasser, monitoring across multiple periods of the year would be necessary (El Gammal et al. 2010). This information would allow us to determine if the correlation between crocodiles and fishermen camps varies accordingly, so that conservation actions could be targeted at the most critical period of the year.

For developing strategies for human–crocodile coexistence, it will further be important to take the fishermen's perspectives into account and determine the nature of the conflicts and to disentangle perceived and actual effects of crocodiles on humans and their livelihood, i.e., fishing activities (Tucker et al. 1996; Platt et al. 2002, 2006, 2013; Wallace and Leslie 2008; Nifong and Silliman 2013; Hanson et al. 2015; Somaweera et al. 2020). Also, understanding crocodile movements will help to identify areas with a lower probability of crocodile encounters where fishermen can place their fishing gear, reducing the encounters and direct damage to the fishing nets (Sai et al. 2016; Shaney et al. 2019; Ouedraogo et al. 2022). Moreover, coexistence between Nile crocodiles and fishermen can be fostered by informing fishermen that Nile crocodiles may not deplete fish stock, but may keep predatory fish species at low densities, allowing the fish species wanted by the fishermen to thrive (Wallace and Leslie 2008).

While our correlative study did not fully disentangle the role of the environmental features and fishermen camp locations, it highlights the existence of a potential conflict, since crocodiles are not driven away by fishing activities. At the same time, the co‐occurrence of Nile crocodiles and fishermen's camps stresses the need to put effort into reducing the conflicts and promoting their coexistence. Thus, it is even more important to monitor the situation and further disentangle the causes of the positive correlation between fishermen and crocodiles found in this study. This way, strategies that lead to fishermen accepting crocodile presence and reducing hunting pressure can be developed (Calverley and Downs 2014; Somaweera et al. 2019, 2020).

In conclusion, this study contributes to our understanding of which environmental and anthropogenic variables determine the distribution of Nile crocodiles. The distance to fishermen's camps as the most important anthropogenic variable showed its impact on the distribution of Nile crocodiles, an aquatic cryptic apex predator species, along Lake Nasser and stresses the need to further investigate the potential conflicts and coexistence between Nile crocodiles and fishermen.

## Author Contributions

**Mohamed A. Ezat:** conceptualization (equal), data curation (equal), funding acquisition (equal), investigation (equal), writing – original draft (equal), writing – review and editing (equal). **Elke Molenaar:** formal analysis (equal), investigation (equal), methodology (equal), software (equal), validation (equal), visualization (equal), writing – original draft (equal), writing – review and editing (equal). **Marc Naguib:** conceptualization (equal), funding acquisition (equal), investigation (equal), methodology (equal), project administration (equal), resources (equal), supervision (equal), writing – original draft (equal), writing – review and editing (equal). **Frank van Langevelde:** conceptualization (equal), funding acquisition (equal), methodology (equal), resources (equal), supervision (equal), writing – original draft (equal), writing – review and editing (equal).

## Conflicts of Interest

The authors declare no conflicts of interest.

## Data Availability

The data underlying this manuscript is available in the 4TU data repository: https://doi.org/10.4121/0e245592‐63a8‐47e7‐b7b8‐0136082caa7e.

## Appendix 1

List of independent variables explaining the distribution of the crocodiles in Lake Nasser, Egypt. The resolution of all variables was set at 30‐arc sec.

**Table:**

No	Category	Variables	Description	Unit	Source
1	Topography	Elevation		m	Worldclim.org
2	Topography	Slope	Based on the elevation data	degree	Worldclim.org
3	Climate	Bio1	Annual mean temperature	Celsius	Worldclim.org
4	Climate	Bio2	Mean diurnal range	Celsius	Worldclim.org
5	Climate	Bio3	Isothermality (Bio2/Bio7)	%	Worldclim.org
6	Climate	Bio4	Temperature seasonality	Celsius	Worldclim.org
7	Climate	Bio5	Max temp. of warmest month	Celsius	Worldclim.org
8	Climate	Bio6	Min temp. of coldest month	Celsius	Worldclim.org
9	Climate	Bio7	Temperature annual range	Celsius	Worldclim.org
10	Climate	Bio8	Mean temp. of wettest quarter	Celsius	Worldclim.org
11	Climate	Bio9	Mean temp. of driest quarter	Celsius	Worldclim.org
12	Climate	Bio10	Mean temp. of warmest quarter	Celsius	Worldclim.org
13	Climate	Bio11	Mean temp. of coldest quarter	Celsius	Worldclim.org
14	Climate	Bio12	Annual precipitation	mm	Worldclim.org
15	Climate	Bio13	Precipitation of wettest month	mm	Worldclim.org
16	Climate	Bio14	Precipitation of driest month	mm	Worldclim.org
17	Climate	Bio15	Precipitation seasonality	Unitless	Worldclim.org
18	Climate	Bio16	Precipitation of wettest quarter	mm	Worldclim.org
19	Climate	Bio17	Precipitation of driest quarter	mm	Worldclim.org
20	Climate	Bio18	Precipitation of warmest quarter	mm	Worldclim.org
21	Climate	Bio19	Precipitation of coldest quarter	mm	Worldclim.org
22	Anthropogenic	Eucl_Distance	Euclidean distance to fishermen camps	km	
23	Anthropogenic	Path_Distance	Path distance to the fishermen camps	km	

## Appendix 2

Estimates of the relative contributions of the environmental and anthropogenic variables in the Maxent model explaining the distribution of the crocodiles in Lake Nasser, Egypt. Note that the percent contribution is sensitive to correlations of the independent variables, whereas the permutation importance is less sensitive.

**Table:**

No	Variables	Percent contribution	Permutation importance
1	Euclidean distance to fishermen camps	30.2	33.2
2	Path Distance to the fishermen camps	26.7	14.1
3	Mean temp. of wettest quarter	11.7	2.6
4	Temperature seasonality	9.9	22.4
5	Slope of the shorelines	7.3	8.2
6	Min temp. of coldest month	6.6	12.8
7	Mean temp. of coldest quarter	2	1
8	Mean temp. of warmest quarter	1.2	0.5
9	Annual Mean Temperature	1.1	0.6
10	Temperature annual range	0.8	3.2
11	Mean temp. of driest quarter	0.7	0
12	Elevation of the shorelines	0.7	0.4
13	Max temp. of warmest month	0.3	0
14	Mean diurnal range	0.3	0
15	Isothermality (Bio2/Bio7)	0.2	0.2
16	Precipitation of warmest quarter	0.1	0.4
17	Precipitation of wettest month	0.1	0.4
18	Precipitation seasonality	0	0
19	Annual precipitation	0	0
20	Precipitation of wettest quarter	0	0
21	Precipitation of coldest quarter	0	0
22	Precipitation of driest quarter	0	0
23	Precipitation of driest month	0	0

## References

[ece371970-bib-0001] Araujo, M. B. , R. G. Pearson , W. Thuiller , and M. Erhard . 2005. “Validation of Species‐Climate Impact Models Under Climate Change.” Global Change Biology 11, no. 9: 1504–1513.

[ece371970-bib-0002] Balaguera‐Reina, S. A. , M. Venegas‐Anaya , A. Sánchez , I. Arbelaez , H. A. Lessios , and L. D. Densmore . 2016. “Spatial Ecology of the American Crocodile in a Tropical Pacific Island in Central America.” PLoS One 11, no. 6: e0157152. 27280554 10.1371/journal.pone.0157152 PMC4900666

[ece371970-bib-0003] Bishai, H. M. , S. A. Abdel‐Malek , and M. T. Khalil . 2000. Lake Nasser. National Biodiversity Unit.

[ece371970-bib-0004] Calverley, P. M. , and C. T. Downs . 2014. “Habitat Use by Nile Crocodiles in Ndumo Game Reserve, South Africa: A Naturally Patchy Environment.” Herpetologica 70, no. 4: 426–438.

[ece371970-bib-0005] Carter, N. H. , and J. D. C. Linnell . 2016. “Co‐Adaptation Is Key to Coexisting With Large Carnivores.” Trends in Ecology & Evolution 31, no. 8: 575–578. 27377600 10.1016/j.tree.2016.05.006

[ece371970-bib-0006] Chakanyuka, T. , and B. Utete . 2022. “Adaptive Co‐Management, Co‐Existence or Just Wildlife Conservation? Case Study of the Human and Nile Crocodile (*Crocodylus niloticus*) Conflicts in Ngezi Dam, Mashonaland West, Zimbabwe.” African Journal of Ecology 60, no. 3: 759–768.

[ece371970-bib-0007] Combrink, X. , J. K. Warner , and C. T. Downs . 2016. “Nest Predation and Maternal Care in the Nile Crocodile ( *Crocodylus niloticus* ) at Lake St Lucia, South Africa.” Behavioural Processes 133: 31–36. 27816524 10.1016/j.beproc.2016.10.014

[ece371970-bib-0008] El Gammal, E. A. , S. M. Salem , and A. E. A. El Gammal . 2010. “Change Detection Studies on the World's Biggest Artificial Lake (Lake Nasser, Egypt).” Egyptian Journal of Remote Sensing and Space Science 13, no. 2: 89–99.

[ece371970-bib-0009] El‐Shirbeny, M. A. , and K. A. Abutaleb . 2018. “Monitoring of Water‐Level Fluctuation of Lake Nasser Using Altimetry Satellite Data.” Earth Systems and Environment 2: 367–375.

[ece371970-bib-0010] Estes, J. A. , J. Terborgh , J. S. Brashares , et al. 2011. “Trophic Downgrading of Planet Earth.” Science 333, no. 6040: 301–306. 21764740 10.1126/science.1205106

[ece371970-bib-0011] Everatt, K. T. , J. F. Moore , and G. I. H. Kerley . 2019. “Africa's Apex Predator, the Lion, Is Limited by Interference and Exploitative Competition With Humans.” Global Ecology and Conservation 20: e00758.

[ece371970-bib-0013] Fick, S. E. , and R. J. Hijmans . 2017. “WorldClim 2: New 1‐Km Spatial Resolution Climate Surfaces for Global Land Areas.” International Journal of Climatology 37, no. 12: 4302–4315.

[ece371970-bib-0014] Focardi, S. , and F. Cagnacci . 2012. Animal Movement. Animal Movement; Statistical Models for Telemetry Data, edited by M. B. Hooten , D. S. Johnson , B. T. McClintock , and J. M. Morales , 259–276. CRC Press.

[ece371970-bib-0015] Graham, C. H. , and R. J. Hijmans . 2006. “A Comparison of Methods for Mapping Species Ranges and Species Richness.” Global Ecology and Biogeography 15, no. 6: 578–587.

[ece371970-bib-0016] Halls, A. , O. A. Habib , A. Nasr‐Allah , and M. Dickson . 2015. Lake Nasser Fisheries: Literature Review and Situation Analysis. WorldFish.

[ece371970-bib-0017] Hanson, J. O. , S. W. Salisbury , H. A. Campbell , R. G. Dwyer , T. D. Jardine , and C. E. Franklin . 2015. “Feeding Across the Food Web: The Interaction Between Diet, Movement and Body Size in Estuarine Crocodiles ( *Crocodylus porosus* ).” Austral Ecology 40, no. 3: 275–286.

[ece371970-bib-0018] Hijmans, R. J. , S. Phillips , J. Leathwick , J. Elith , and M. R. J. Hijmans . 2017. “Package ‘Dismo’.” Circles 9, no. 1: 1–68.

[ece371970-bib-0019] Hill, R. , G. J. W. Webb , and A. M. A. Smith . 1987. “Floating Vegetation Mats on a Floodplain Billabong in the Northern Territory of Australia.” Hydrobiologia 150, no. 2: 153–164.

[ece371970-bib-0020] Hu, W. , Y. Wang , P. Dong , et al. 2020. “Predicting Potential Mangrove Distributions at the Global Northern Distribution Margin Using an Ecological Niche Model: Determining Conservation and Reforestation Involvement.” Forest Ecology and Management 478: 118517.

[ece371970-bib-0021] Hutton, J. M. , and M. E. J. Woolhouse . 1989. “Mark‐Recapture to Assess Factors Affecting the Proportion of a Nile Crocodile Population Seen During Spotlight Counts at Ngezi, Zimbabwe, and the Use of Spotlight Counts to Monitor Crocodile Abundance.” Journal of Applied Ecology 26, no. 2: 381.

[ece371970-bib-0022] Isberg, S. , X. Combrink , C. Lippai , and S. A. Balaguera‐Reina . 2019. Crocodylus niloticus. IUCN Red List of Threatened Species.

[ece371970-bib-0023] König, H. J. , C. Kiffner , S. Kramer‐Schadt , C. Fürst , O. Keuling , and A. T. Ford . 2020. “Human–Wildlife Coexistence in a Changing World.” Conservation Biology 34, no. 4: 786–794. 32406977 10.1111/cobi.13513

[ece371970-bib-0024] Levy, C. 1991. Crocodiles and Alligators. Book Sales.

[ece371970-bib-0025] Mazzotti, F. J. , G. R. Best , L. A. Brandt , M. S. Cherkiss , B. M. Jeffery , and K. G. Rice . 2009. “Alligators and Crocodiles as Indicators for Restoration of Everglades Ecosystems.” Ecological Indicators 9, no. 6: S137–S149.

[ece371970-bib-0027] Montero, J. R. B. , J. J. S. Ramírez , L. Sigler , B. R. Barr , and I. S. Hernández . 2019. “Population Status of the American Crocodile, *Crocodylus Acutus* (Reptilia: Crocodilidae) and the Caiman, *Caiman Crocodilus* (Reptilia: Alligatoridae), in the Central Caribbean of Costa Rica.” Revista de Biología Tropical 67, no. 6: 1180–1193.

[ece371970-bib-0028] Nifong, J. C. , and B. R. Silliman . 2013. “Impacts of a Large‐Bodied, Apex Predator ( *Alligator mississippiensis* Daudin 1801) on Salt Marsh Food Webs.” Journal of Experimental Marine Biology and Ecology 440: 185–191.

[ece371970-bib-0032] Ouedraogo, I. , A. Oueda , M. E. Hema , M. H. Shirley , and B. G. Kabre . 2022. “Impact of Anthropogenic Activities on the Abundance of (Crocodylus Suchus) (Saint‐Hilaire 1807) Within the Nazinga Game Ranch, Burkina Faso.” Open Journal of Ecology 12, no. 12: 788–803.

[ece371970-bib-0033] Parker, I. S. C. 1970. “Crocodile Distribution and Status in the Major Waters of Western and Central Uganda in 1969.” African Journal of Ecology 8, no. 1: 85–103.

[ece371970-bib-0034] Phillips, S. J. , R. P. Anderson , and R. E. Schapire . 2006. “Maximum Entropy Modeling of Species Geographic Distributions.” Ecological Modelling 190, no. 3: 231–259.

[ece371970-bib-0035] Platt, S. G. , T. R. Rainwater , and S. T. McMurry . 2002. “Diet, Gastrolith Acquisition and Initiation of Feeding Among Hatchling Morelet's Crocodiles in Belize.” Herpetological Journal 12, no. 2: 81–84.

[ece371970-bib-0036] Platt, S. G. , J. B. Thorbjarnarson , T. R. Rainwater , and D. R. Martin . 2013. “Diet of the American Crocodile ( *Crocodylus acutus* ) in Marine Environments of Coastal Belize.” Journal of Herpetology 47, no. 1: 1–10.

[ece371970-bib-0037] Platt, S. G. S. G. , T. R. T. R. Rainwater , A. G. A. G. Finger , J. B. J. B. Thorbjarnarson , T. A. T. A. Anderson , and S. T. S. T. McMurry . 2006. “Food Habits, Ontogenetic Dietary Partitioning and Observations of Foraging Behaviour of Morelet's Crocodile (*Crocodylus moreletii*) in Northern Belize.” Herpetological Journal 16, no. 3: 281–290.

[ece371970-bib-0038] Pooley, S. 2015. “Using Predator Attack Data to Save Lives, Human and Crocodilian.” Oryx 49, no. 4: 581–583.

[ece371970-bib-0039] Ripple, W. J. , J. A. Estes , R. L. Beschta , et al. 2014. “Status and Ecological Effects of the World's Largest Carnivores.” Science 343, no. 6167: 1241484. 24408439 10.1126/science.1241484

[ece371970-bib-0040] Sai, M. , B. Utete , E. Chinoitezvi , G. H. Moyo , and E. Gandiwa . 2016. “A Survey of the Abundance, Population Structure, and Distribution of Nile Crocodiles (*Crocodylus Niloticus*) Using Day Ground Surveys in Sengwa Wildlife Research Area, Zimbabwe.” Herpetological Conservation and Biology 11, no. 3: 426–433.

[ece371970-bib-0041] Shaney, K. J. , A. Hamidy , M. Walsh , E. Arida , A. Arimbi , and E. N. Smith . 2019. “Impacts of Anthropogenic Pressures on the Contemporary Biogeography of Threatened Crocodilians in Indonesia.” Oryx 53, no. 3: 570–581.

[ece371970-bib-0042] Shirley, M. H. , R. M. Dorazio , E. Abassery , A. A. Elhady , M. S. Mekki , and H. H. Asran . 2012. “A Sampling Design and Model for Estimating Abundance of Nile Crocodiles While Accounting for Heterogeneity of Detectability of Multiple Observers.” Population Ecology 76, no. 5: 966–975.

[ece371970-bib-0043] Somaweera, R. , M. L. Brien , T. Sonneman , R. K. Didham , and B. L. Webber . 2019. “Absence of Evidence Is Not Evidence of Absence: Knowledge Shortfalls Threaten the Effective Conservation of Freshwater Crocodiles.” Global Ecology and Conservation 20: e00773.

[ece371970-bib-0044] Somaweera, R. , J. Nifong , A. Rosenblatt , et al. 2020. “The Ecological Importance of Crocodylians: Towards Evidence‐Based Justification for Their Conservation.” Biological Reviews 95, no. 4: 936–959. 32154985 10.1111/brv.12594

[ece371970-bib-0046] Treves, A. , and K. U. Karanth . 2003. “Human‐Carnivore Conflict and Perspectives on Carnivore Management Worldwide.” Conservation Biology 17, no. 6: 1491–1499.

[ece371970-bib-0047] Tucker, A. D. , C. J. Limpus , H. I. McCallum , and K. R. McDonald . 1996. “Ontogenetic Dietary Partitioning by *Crocodylus johnstoni* During the Dry Season.” Copeia 1996, no. 4: 978.

[ece371970-bib-0050] Wallace, K. M. , and A. J. Leslie . 2008. “Diet of the Nile Crocodile ( *Crocodylus niloticus* ) in the Okavango Delta, Botswana.” Journal of Herpetology 42, no. 2: 361–368.

[ece371970-bib-0052] Woodroffe, R. 2000. “Predators and People: Using Human Densities to Interpret Declines of Large Carnivores Animal Conservation Forum.” Cambridge Core 3: 165–173.

